# Germline and Somatic *BRCA1/2* Mutations in 172 Chinese Women With Epithelial Ovarian Cancer

**DOI:** 10.3389/fonc.2020.00295

**Published:** 2020-03-10

**Authors:** Yan You, Lei Li, Junliang Lu, Huanwen Wu, Jing Wang, Jie Gao, Ming Wu, Zhiyong Liang

**Affiliations:** ^1^Department of Pathology, Molecular Pathology Research Center, Peking Union Medical College Hospital, Peking Union Medical College and Chinese Academy of Medical Science, Beijing, China; ^2^Department of Obstetrics and Gynecology, Peking Union Medical College Hospital, Peking Union Medical College and Chinese Academy of Medical Science, Beijing, China

**Keywords:** epithelial ovarian cancer, *BRCA* mutations, germline mutations, somatic mutations, progression-free survival, overall survival

## Abstract

**Objective:** Despite several nationwide cohort studies of germline *BRCA1/2* mutations and several small cohort studies of somatic *BRCA1/2* mutations in Chinese epithelial ovarian cancer (EOC) patients, little is known about the impact of these findings on survival outcomes in this population. In this study of 172 retrospectively recruited Chinese EOC patients, germline and somatic *BRCA1/2* mutations and their value for predicting survival outcomes were evaluated.

**Methods:** Unselected patients who visited the study center from January 1, 2011, to January 1, 2015, were recruited and asked to provide peripheral blood samples for this study if they were pathologically confirmed to have primary EOC. All patients received staging surgeries or debulking surgeries involving systemic platinum-based chemotherapy, and the patients were then followed up to December 1, 2017. DNA was extracted from formalin-fixed, paraffin-embedded (FFPE) sections and peripheral blood and sequenced for somatic and germline testing, respectively. The demographic and clinicopathological characteristics of the patients were collected to analyze the distribution of *BRCA* mutations in subgroups. Survival outcomes were compared among various *BRCA* mutation statuses using univariate and multivariate models.

**Results:** In 58 (33.7%) patients, 63 variants were identified, including variants of unknown significance (VUS) in 18 patients (10.5%) and pathogenic or likely pathogenic variants in a partially overlapping set of 41 patients (23.8%). Germline *BRCA* mutations, somatic *BRCA* mutations, *BRCA1* mutations in general, and *BRCA2* mutations in general were found in 35 (20.3%), 7 (4.1%), 28 (16.3%), and 13 (7.6%) patients, respectively. Five recurrent mutations were identified. Personal and family cancer histories as well as hereditary breast and ovarian cancer (HBOC) criteria were associated with deleterious *BRCA* mutations both overall and in the germline specifically, whereas only age at diagnosis of EOC was associated with somatic *BRCA* mutations. In univariate and Cox regression analyses, patients with *BRCA1/2* mutations in general had significant improvements in progression-free survival (PFS) and overall survival (OS).

**Conclusions:** In Chinese EOC patients, the distributions and risk factors associated with germline and somatic *BRCA1/2* mutations were similar to those previously reported in international studies. Deleterious *BRCA* mutations in general were associated with improved survival outcomes in this cohort.

## Introduction

Ovarian cancer is the third most common gynecological malignancy and the leading cause of mortality in female cancers (1), representing 1.3% of all new cancer cases in the United States in 2018 (2). In China, the prevalence of ovarian cancer has increased in the past decade, with 52,100 new cases and 22,500 related deaths in 2015 (3). Ovarian cancers are a heterogeneous group of malignancies varying in etiology and molecular biology. Approximately 90% of cases belong to the epithelial type [epithelial ovarian cancer (EOC)], with the most common being high-grade serous carcinoma (HGSC). A majority of EOC patients are diagnosed at advanced stages and have a poor prognosis. As with other malignancies, the tumorigenesis of EOC is a process that drives normal cells toward a malignant state and can involve both somatic (acquired) and germline (inherited) mutations (4, 5). Large-scale cancer sequencing data from cases in The Cancer Genome Atlas (TCGA) revealed that ovarian cancer has the highest prevalence of susceptibility-associated genes (6). In previous reports, ~5 to 10% of invasive EOC cases were hereditary (7–10). In addition, inherited ovarian cancer may present as hereditary breast and ovarian cancer (HBOC) (11, 12). However, in recent reports, ~20% or more of all EOCs have been identified to be associated with germline mutations (13–16). Most cases of inherited susceptibility to EOC are primarily related to germline mutations of *BRCA1* and *BRCA2*, which account for about 80% of hereditary ovarian cancers (17–19). Mutant *BRCA* is an indispensable founding mutation in EOC (20). Knowledge of the pathogenic molecular mechanism and genetic mutations involved in EOC has promoted genetic counseling and testing as well as potential intervention (21). The emergence of poly(adenosine diphosphate [ADP]-ribose) polymerase inhibitors (PARPis) has necessitated genetic testing (22). However, there is controversy regarding the optimum testing strategy (23–25). Information on *BRCA1/2* germline mutations has predictive value for the platinum sensitivity of tumors and the survival outcomes of patients (26–28). In a study by Pennington et al. (28) compared to germline *BRCA1/2* mutations, somatic *BRCA1/2* mutations had a similar positive impact on overall survival (OS) and platinum responsiveness. On the other hand, in recent reports, homologous recombination deficiency has gained in importance, in addition to the BRCA mutations, on the targeted treatment (23, 29), chemotherapy (30, 31), and prognosis (32, 33) in EOC patients.

Despite several national cohort studies of *BRCA* germline mutations in China (16, 34, 35) and in Japan (24), no study has attempted to reveal the association between mutations and treatment effects or survival outcomes in Chinese EOC patients. The specific impact of somatic mutations on EOC cohorts is also not well-explored. In our previous report (36), no significant outcomes were discovered due to the limited sample size and short follow-up period.

In the present report, we aimed to determine the frequencies of germline and somatic *BRCA1/2* mutations in a single study center based on pathological findings from primary samples or surgical tissues. The mutation status was analyzed in the context of various demographic and clinicopathological characteristics, such as personal and family cancer history as well as histological subtypes. The impact of mutation status on sensitivity to platinum-based chemotherapy and on patient survival outcomes was also described in univariate and multivariate models.

## Materials and Methods

### Study Subjects

Unselected patients who visited the study center from January 1, 2011, to January 1, 2015, and were diagnosed with EOC were recruited for this study. Once the pathological examination confirmed EOC, the patients were asked to provide samples of peripheral blood for germline testing if they met the following inclusion criteria: (1) age 18 years or older; (2) pathologically confirmed EOC (including EOC, carcinoma of the fallopian tube and primary peritoneal carcinoma); (3) receipt of comprehensive staging surgery or debulking surgery and systemic platinum-based chemotherapy; (4) sufficient formalin-fixed, paraffin-embedded (FFPE) sections for somatic testing, which could be performed between any two systematic chemotherapy treatments; these tissues could be collected by core biopsy or by laparoscopy for patients prepared for neoadjuvant chemotherapy or by laparotomic sampling for patients of primary staging or cytoreductive surgeries; (5) willingness to provide signed consent in advance of the trial; and (6) provision of peripheral blood for germline testing. Patients not meeting all of these inclusion criteria were excluded. Basic patient information regarding age at diagnosis, neoplasm staging and histopathological type was retrieved from medical records. This study was approved by the Institutional Review Board (IRB) of the study center (Registration No. ZS-1245). The Chinese Human Genetic Resources Management Office of the National Ministry of Science and Technology also approved this study (registration No. [2017]1901, http://www.most.gov.cn/bszn/new/rlyc/jgcx/index.htm). As previously mentioned, all patients gave informed consent before enrollment.

### Treatment and Follow-Up

All patients received staging surgeries or primary or interval debulking surgeries along with systematic chemotherapy, which was initiated with platinum-based chemotherapy. Demographic data and medical history, including the date of diagnosis, were retrieved from the patients' medical records. Data regarding histological classification and grading were obtained from the electronic pathology database at the study center. Staging was determined by the International Federation of Gynecology and Obstetrics (FIGO) 2014 staging system (37). Personal and family cancer histories were addressed, especially personal breast cancer history and the aspects of cancer history specified by the “Criteria for further genetic risk evaluation” from the National Comprehensive Cancer Network (NCCN) HBOC guideline of “Genetic/Familial High-Risk Assessment: Breast and Ovarian” (38).

Tumors were sorted into the following categories depending on their response to platinum-based chemotherapy: (1) platinum sensitive, if the time between administration and first relapse of the disease was more than 6 months; (2) platinum resistant, if the patient had a relapse 4 weeks to 6 months after administration; or (3) refractory, if the disease relapsed within 4 weeks under platinum-based agents. Follow-ups were conducted for all enrolled patients until December 1, 2017, or death. Disease relapse or progression was determined by medical imaging, serology, or histology. Progression-free survival (PFS) was defined as the time span between initial diagnosis and disease relapse or progression, while OS was defined as the time span between initial diagnosis and patient death.

### Analysis of Germline and Somatic *BRCA1/2* Mutational Status

For germline *BRCA1/2* analyses, DNA was extracted from 200 μl of peripheral blood with the QIAamp DNA Blood Mini Kit (QIAGEN, Hilden, Germany). For somatic *BRCA1/2* analysis, 10 FFPE sections measuring five micrometers in thickness and taken from representative blocks were made for each case. The cancerous region was then marked by two experienced pathologists (YY and HW) and macro-dissected following deparaffinization using xylene. DNA was then extracted from the tumor tissue using the QIAamp DNA FFPE Tissue Kit (QIAGEN, Hilden, Germany).

Sequencing libraries were prepared using an Oncomine *BRCA* research assay (Thermo Fisher, Waltham, Massachusetts, USA); the libraries were then subjected to emulsion PCR using the Ion OneTouch 2 System and sequenced on an Ion Torrent Personal Genome Machine sequencer to a median of 500X depth for germline mutations and 1000X for somatic mutations according to the manufacturer's instructions. Upon completion, the raw data were sent through a pipeline customized by Life Technologies. The parameters for variation calling are available (in JSON format) upon request. The sequences covered all exons of the *BRCA1* and *BRCA2* genes as well as ±20 bp flanking regions to enable the detection of variations affecting potential splice sites. However, due to its high cost, we did not include multiplex ligation-dependent probe amplification (MLPA) in the tests for *BRCA1/2*.

The identified *BRCA1/2* variations were classified according to the 2015 American College of Medical Genetics and Genomics (ACMG) guidelines (39). Pathogenic and likely pathogenic mutations were regarded as deleterious mutations.

### Statistical Analysis

Comparisons of continuous variables were conducted with parametric methods if the assumption of normality was met. Non-normally distributed variables and categorical data were compared between patients with and without specific deleterious mutations by using non-parametric tests. Kaplan–Meier survival curves were generated, and proportional hazards models were used to estimate the hazard ratios (HRs) and 95% confidence intervals (95% CIs) for the effects of deleterious mutations on PFS and OS. A multivariable analysis of disease-free survival was performed with adjustment for the important baseline risk factors of major histological subtype (HGSC vs. others), age at diagnosis, and stage (stage I–II vs. III–IV). Unless otherwise stated, all analyses used a two-sided significance level of 0.05 and were conducted with Statistical Product and Service Solutions (SPSS) Statistics 20.0 software (IBM Corporation, Armonk, NY, USA).

## Results

### Demographic and Clinicopathological Characteristics of Ovarian Cancer Patients

The demographic and clinicopathological characteristics of the patients are listed in Table 1. In total, 172 patients were recruited and had final genetic testing outcomes. The median age at diagnosis in the cohort was 52.5 years (range 18–81). Most cases were ovarian carcinomas (168 cases, 97.7%), stage III-IV (140 cases, 81.4%), and HGSC (138 cases, 80.2%). Based on personal and family cancer history, 31 patients met the HBOC criteria. No patients had ever received PARPi treatment. Progression and mortality occurred in 106 (61.6%) and 50 (29.1%) patients, and the median PFS and OS were 23 months (range 0–68) and 39 (12–93) months, respectively.

**Table 1:** The clinicopathological characteristics and survival outcomes of the patients.

Age at diagnosis of EOC (years), median (range)	52.5 (18–81)
**Cancer sites, *n* (%)**	
Ovarian carcinoma	168 (97.7%)
Fallopian tube carcinoma	3 (1.7%)
Primary peritoneal carcinoma	1 (0.6%)
**FIGO stages, *n* (%)**	
Stage I	19 (11.0%)
Stage II	12 (7.0%)
Stage III	122 (69.8%)
Stage IV	18 (10.5%)
Not specific	1 (0.6%)
Histological subtypes, *n* (%)^*^	
HGSC	138 (80.2%)
Clear cell	9 (5.2%)
Endometrioid	10 (5.8%)
LGSC	4 (2.3%)
Mucinous	4 (2.3%)
Squamous	1 (0.6%)
Brener	2 (1.2%)
Carcinosarcoma	1 (0.6%)
Undifferentiated	1 (0.6%)
Unspecific	2 (1.2%)
Personal cancer history before the diagnosis of EOC, *n* (%)	13 (7.6%)
Personal breast cancer history, *n* (%)	8 (4.7%)
Family cancer history, *n* (%)	67 (39.0%)
Cancer history according with HBOC criteria, *n* (%)^†^	31 (18.0%)
**Sensitivity to platinum-based chemotherapy**	
Sensitive, *n* (%)	141 (82.2%)
Resistant, *n* (%)	22 (12.8%)
Refractory, *n* (%)	9 (5.2%)
Recurrence, *n* (%)	106 (61.6%)
PFS (months), median (range)	23 (0–68)
Death, *n* (%)	50 (29.1%)
OS (months), median (range)	39 (12–93)

*EOC, epithelial ovarian cancer; HBOC, hereditary breast and ovarian cancer; HGSC, high-grade serous carcinoma; LGSC, low-grade serous carcinoma; OS, overall survival; PFS, progression-free survival; SD, standard deviation*.

* *For 2 cases of unspecific histological subtype, pathological review for the biopsy specimens only discovered carcinomas arising from the epithelium of Mullerian tubes. However, after neoajuvant chemotherapy, interval debulking surgeries in these 2 cases didn't provide tumor specimens for further classifications*.

† *HBOC criteria were based on the “Criteria for further genetic risk evaluation” from NCCN Clinical Practice Guidelines in Oncology (NCCN Guidelines) (30)*.

### Mutation Analysis

**Figure 1:**
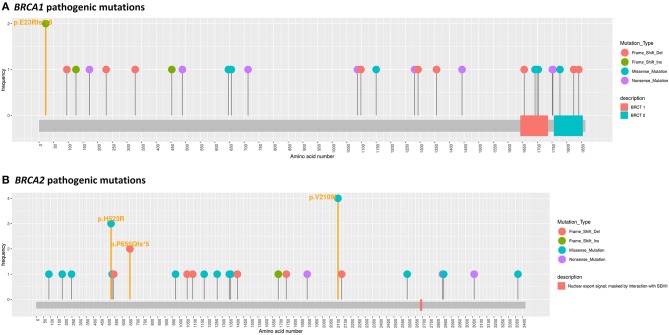
*BRCA1* **(A)** and *BRCA2* **(B)** pathogenic mutation loci.

In 58 (33.7%) patients, 63 variants were identified, including variants of unknown significance (VUS) in 18 patients (10.5%) and pathogenic or likely pathogenic variants in a partially overlapping set of 41 patients (23.8%) (Figure 1). Germline *BRCA* mutations, somatic *BRCA* mutations, BRCA1 mutations in general, and *BRCA2* mutations in general were found in 35 (20.3%), 7 (4.1%), 28 (16.3%), and 13 (7.6%) patients, respectively. The mutation details are listed in Supplement Table 1. Likely pathogenic or pathogenic mutations consisted of 19 (46.3%) frameshift deletions, 4 (9.8%) frameshift insertions, 12 (29.3%) non-sense mutations, 4 (9.8%) splice-site mutations, and 2 (4.9%) missense mutations.

As shown in Table 2 and Supplement Table 1, 35 (19.7%) and seven (4.1%) patients had at least one germline and somatic deleterious mutation, respectively; 28 (16.3%) and 13 (7.6%) patients harbored at least one deleterious mutation of *BRCA1* and *BRCA2*, respectively. Two (1.2%), 5 (2.9%), and 2 (1.2%) patients carried dual germline/somatic mutations; dual germline and dual somatic mutations classified as VUS; and dual likely pathogenic and pathogenic mutations, respectively. In cases No. 063 and No. 068, the distance between the co-occurring mutations exceeded the amplicon length; therefore, we were unable to determine whether these mutations were in cis or in trans. The criteria for reclassification were unmet for these mutations. In case No. 032, the patient harbored one likely pathogenic germline variant and one somatic pathogenic variant (Supplement Table 1).

**Table 2:** Distributions of pathogenic or likely pathogenic mutations in the overall and subgroup populations.

	**All *BRCA1/2*, *n* (%)**	**Germline *BRCA1/2*, *n* (%)**	**Somatic *BRCA1/2*, *n* (%)**	***BRCA1*, *n* (%)**	***BRCA2*, *n* (%)**
Whole population (*n* = 172)	41 (23.8%)	35 (20.3%)	7 (4.1%)	28 (16.3%)	13 (7.6%)
**Various subgroups**					
Age at diagnosis of EOC					
<50 years (*n* = 63)	12 (19.0%)	12 (19.0%)	0 (0.0%)	10 (15.9%)	2 (3.2%)
>50 years (*n* = 109)	29 (26.6%)	23 (21.1%)	7 (6.4%)	18 (16.5%)	11 (10.1%)
*p*-values	0.262	0.747	0.048	0.913	0.083
FIGO stages					
Stage I–II (*n* = 31)	6 (19.4%)	5 (16.1%)	1 (3.2%)	6 (19.4%)	0 (0.0%)
Stage III–IV (*n* = 141)	35 (24.8%)	30 (21.3%)	6 (4.3%)	22 (15.6%)	13 (9.2%)
*p*-values	0.518	0.519	0.631	0.608	0.068
Histological subtypes					
HGSC (*n* = 138)	38 (27.5%)	32 (23.2%)	7 (5.1%)	27 (19.6%)	11 (8.0%)
Others (*n* = 34)	3 (8.8%)	3 (8.8%)	0 (0.0%)	1 (2.9%)	2 (5.9%)
*p*-values	0.022	0.062	0.207	0.019	0.506
Personal cancer history					
No (*n* = 159)	34 (21.4%)	28 (17.6%)	7 (4.4%)	22 (13.8%)	12 (7.5%)
Yes (*n* = 13)	7 (53.8%)	7 (53.8%)	0 (0.0%)	6 (46.2%)	1 (7.7%)
*p*-values	0.015	0.002	0.571	0.008	0.654
Personal breast cancer history					
No (*n* = 164)	34 (20.7%)	28 (17.1%)	7 (4.3%)	22 (13.4%)	12 (7.3%)
Yes (*n* = 8)	7 (87.5%%)	7 (87.5%)	0 (0.0%)	6 (75.0%)	1 (12.5%)
*p*-values	<0.001	<0.001	0.712	<0.001	0.474
Family cancer history					
No (*n* = 105)	17 (16.2%)	11 (10.5%)	6 (5.7%)	10 (9.5%)	7 (6.7%)
Yes (*n* = 67)	24 (35.8%)	24 (35.8%)	1 (1.5%)	18 (26.9%)	6 (9.0%)
*p*-values	0.003	<0.001	0.167	0.003	0.580
HBOC criteria^*^					
No (*n* = 141)	25 (17.7%)	19 (13.5%)	6 (4.3%)	17 (12.1%)	8 (5.7%)
Yes (*n* = 31)	16 (51.6%)	16 (51.6%)	1 (3.2%)	11 (35.5%)	5 (16.1%)
*p*-values	<0.001	<0.001	0.631	0.001	0.046
Sensitivity to chemotherapy					
Sensitive (*n* = 141)	38 (27.0%)	32 (22.7%)	7 (5.0%)	26 (18.4%)	12 (8.5%)
Resistant or refractory (*n* = 31)	3 (9.7%)	3 (9.7%)	0 (0.0%)	2 (6.5%)	1 (3.2%)
*p*-values	0.041	0.103	0.242	0.102	0.280

*EOC, epithelial ovarian cancer; HBOC, hereditary breast and ovarian cancer; HGSC, high-grade serous carcinoma; LGSC, low-grade serous carcinoma*.

* *HBOC criteria were based on the NCCN guidelines*.

Five recurrent mutations were identified in the cohort. Three pathogenic mutations occurred in two patients (*BRCA1* c.5470_5477delATTGGGCA, *BRCA1* c.66dup, and *BRCA2* c.1963delC). Two VUS missense mutations (*BRCA2* c.1568A>G and *BRCA2* c.6325G>A) appeared in three and four patients, respectively. All recurrent mutations were in the germline except for *BRCA2* c.1964delC.

### Deleterious *BRCA* Mutations and Clinicopathological Characteristics

As shown in Table 2, FIGO stages had no significant association with deleterious mutations of *BRCA* genes in general or of *BRCA1* or *BRCA2* in particular. Personal and family cancer history and HBOC criteria were associated with overall and germline *BRCA* mutations. HBOC criteria were also associated with overall *BRCA1* and *BRCA2* mutations. For somatic *BRCA* mutations, only age at diagnosis had a significant impact, while for germline *BRCA* mutations, histological subtype also had a significant impact. However, only overall deleterious *BRCA* mutations had a significant impact on sensitivity to platinum-based chemotherapy (Table 2).

### Survival Analyses

**Figure 2:**
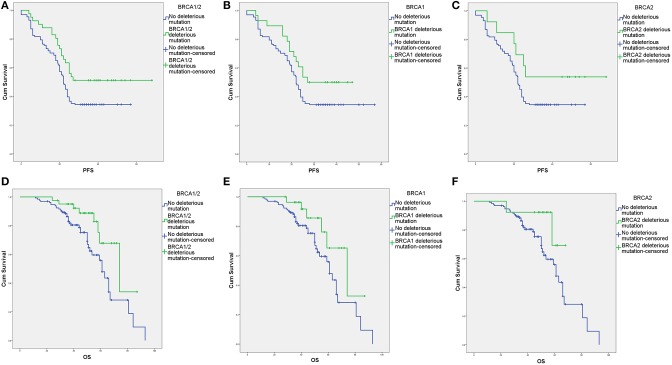
Survival outcomes of various *BRCA* aberrations according to Kaplan–Meier analysis. **(A)** Progression-free survival (PFS) with and without deleterious *BRCA1/2* mutations. **(B)** PFS with and without deleterious *BRCA1* mutations. **(C)** PFS with and without deleterious *BRCA2* mutations. **(D)** Overall survival (OS) with and without deleterious *BRCA1/2* mutations. **(E)** OS with and without deleterious *BRCA1* mutations. **(F)** OS with and without deleterious *BRCA2* mutations.

**Figure 3:**
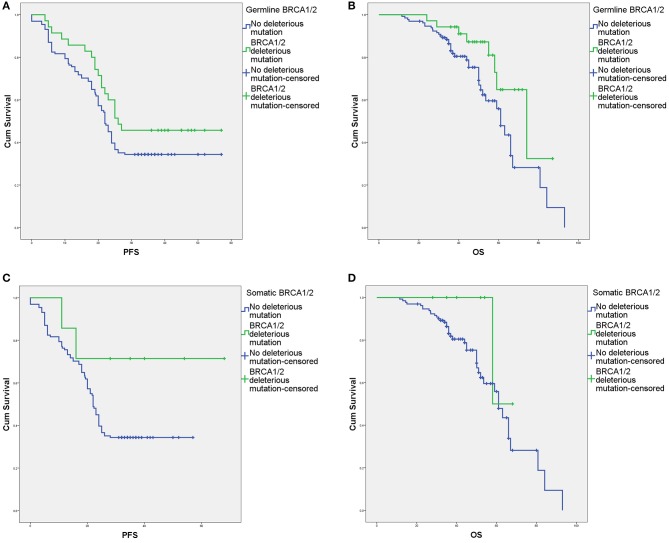
Survival outcomes of germline and somatic *BRCA* aberrations according to Kaplan–Meier analysis. **(A)** Progression-free survival (PFS) with and without deleterious germline *BRCA1/2* mutations. **(B)** Overall survival (OS) with and without deleterious germline *BRCA1/2* mutations. **(C)** PFS with and without deleterious somatic *BRCA1/2* mutations. **(D)** OS with and without deleterious somatic *BRCA1/2* mutations.

The survival outcomes associated with various deleterious *BRCA* mutations are shown in Figures 2, 3 and Supplement Table 2. In univariate and Cox regression analyses, patients with overall *BRCA1/2* mutations had significant improvements in PFS and OS (Figure 2, Supplement Table 2). Overall, germline *BRCA1/2* mutations were associated with significant improvement in OS but not PFS. Overall, *BRCA2* mutations were associated with significantly improved OS in the Cox regression model. Somatic *BRCA1/2* and overall *BRCA1* had no significant impact on survival outcomes. However, in the subgroup analysis of HGSC patients (*n* = 138), various mutation statuses had no significant impact on survival outcomes, except that overall *BRCA* mutations affected PFS (Supplement Table 2).

## Discussion

To our knowledge, this study is the first to describe the positive impact of *BRCA* mutation status on survival outcomes in a Chinese EOC cohort. In our study, overall *BRCA* mutations, germline *BRCA* mutations, and overall *BRCA2* mutations were all associated with improved survival outcomes, as in previous reports (27, 28, 40–42). We did not find a significant impact of germline mutations on PFS or a significant impact of somatic mutations on PFS or OS. The limited sample size was probably the main reason. As shown in Table 2, HGSC patients harbored more deleterious *BRCA1/2* and *BRCA1* mutations than patients with other subtypes, and the BRCAness phenotype was independently associated with improved OS in HGSC patients (43). However, in our study, the effects of sample size were so obvious that HGSC patients, the majority of the cohort, had no significant associations between mutation status and survival outcome (Supplement Table 2). A larger cohort of Chinese EOC patients (NCT03015376) would provide more details and profound perspectives on such issues. The improved survival outcomes were likely caused by a higher sensitivity to platinum in patients with deleterious mutations than in those without such mutations, as revealed in the Pennington et al. (28) study.

Overall, we found that germline and somatic *BRCA1/2* mutations had prevalence rates of 20.3 and 4.1%, respectively. Disparities in both germline and somatic testing exist (44, 45). Regarding other Chinese studies, our germline mutation prevalence approximated the values reported by Li et al. (1331 cases, 22.4%) (16). Wang et al. (481 cases, 19.6%) (46), Shi et al. (916 cases, 17.0%), and a meta-analysis (21.8%) (47), but it was significantly lower than the value reported by Wu et al. (826 cases, 28.4%) (35). Our somatic *BRCA1/2* mutation prevalence was similar to those in two small cohort studies, which reported prevalence values of 4.0% (2/50) (48) and 6.4% (4/62) (36). These differences may originate from the selection of subjects and the database used for analysis. As addressed in a recently published meta-analysis (47), the variant profile in ethnically Chinese people was distinctive from those in other ethnic groups with no distinct founder pathogenic variants. However, this conclusion should be treated cautiously. China needs a uniform platform such as TCGA to further promote genomic testing. Chinese healthcare providers, including gynecologists, still have greatly divergent opinions and suggestions about genetic testing for gynecologic oncology, and these positions are also significantly different from the requirements and recommendations in the guidelines and consensus statements (49–51).

There are several issues worth considering in relation to the selection bias in this report. In our study, HGSC had a significantly higher frequency of overall *BRCA1/2* and *BRCA1* mutations than other subtypes. These tumors displayed defective homologous recombination due to germline and somatic *BRCA* mutations, epigenetic inactivation of *BRCA* and abnormalities in DNA repair genes (9, 13, 14). The quality of the data that can be obtained from FFPE tissues as opposed to fresh tissues needs clarification. The fact that the distribution of somatic mutations was consistent with other Chinese studies (36, 48) and with a Polish study using FFPE tissues from HGSC patients (52) could guarantee the quality of our sample preparation. It was suggested that an adjusted targeted capture-based enrichment protocol was superior to commonly applied multiplex PCR-based protocols for reliable *BRCA1/2* variant detection, including CNV detection, using FFPE tumor samples (53). As previously reported, the prevalence of *BRCA1/2* mutations in patients with peritoneal carcinoma or fallopian tube carcinoma is comparable to that in EOC patients (54). Therefore, we did not perform a subgroup analysis of these three site-specific cancers. We also did not evaluate the impact of neoadjuvant therapy on prognosis by *BRCA* status since primary debulking surgery seems to ensure a longer PFS than neoadjuvant chemotherapy alone in women with the *BRCA* wild-type genotype (55). However, patients with pathogenic mutations in *BRCA* may benefit from intraperitoneal therapy (56). Finally, no patients in our study had ever utilized PARPis. The high cost of genetic testing is an important barrier to universal performance. However, somatic and germline mutations and expression loss of *BRCA1/2* are sufficiently common in ovarian cancer to warrant assessment for the prediction of treatment benefit in clinical trials of PARPis (57).

We reported the previously unknown association between *BRCA* mutation and survival outcomes in Chinese EOC patients, which was the most important strength of our study. However, the present study has several limitations. We did not exploit all mechanisms of *BRCA1/2* regulation, such as large gene rearrangements and epigenetic modifications. Second, the present study population did not include a sufficient sample of histological subtypes other than HGSC; this limitation impairs the ability to capture the mutational landscape of these tumors. Third, as reported previously (58–61). a multigene panel for germline and/or somatic testing would probably be cost effective, providing a substantial rate of clinically actionable pathogenic variants (62). There is a potential benefit to be gained from rescreening with a multigene panel in patients who previously underwent non-informative genetic screening (63). On the other hand, up to 50% of ovarian HGSC patients may exhibit homologous recombination defects (64), which are associated with the indication of PARPis as maintenance therapy, even in newly diagnosed EOC patients (23, 25, 29). These advancements in genetic and genomic testing in EOC will promote further exploration in the future. Importantly, we did not include the MLPA analysis in our study, which is also very important for the evaluation of BRCAness (43, 65). The lack of MLPA obviously decreases the deleterious mutation frequency in patients with EOC or breast cancer and causes bias regarding the association between BRCAness and survival prognosis (66). We did not include *BRCA* methylation in the study. However, unlike *BRCA* mutation, *BRCA1* methylation was not associated with improved survival or greater sensitivity to platinum chemotherapy (67). Last but not least, a discussion of cosegregation analysis and potential interventions (such as NCT03294343 and NCT04190667) should be provided to all patients with deleterious mutations.

## Conclusions

In conclusion, we reported the prevalence of germline and somatic *BRCA1/2* mutations in an unselected Chinese EOC cohort comprising 172 patients. Germline mutations were associated with cancer history, especially the aspects relevant to the HBOC criteria. Somatic mutations were associated only with age at diagnosis of EOC. In our study, overall deleterious *BRCA* mutations predicted increased sensitivity to platinum-based chemotherapy and significantly improved survival outcomes. A larger cohort must be examined with multigene panel testing and even the homologous recombination deficiency model to clarify the indications for PARPis.

## Data Availability Statement

All datasets generated for this study are included in the article/Supplementary Material.

## Ethics Statement

The Institutional Review Board of Peking Union Medical College Hospital approved this study (No. HS-1245). In addition, the Chinese Human Genetic Resources Management Office of the National Ministry of Science and Technology approved this study (registration no. [2017]1901, http://www.most.gov.cn/bszn/new/rlyc/jgcx/index.htm).

## Author Contributions

MW and ZL conceived of the original idea for the study, interpreted the results, carried out the statistical analysis, edited the paper, and acted as overall guarantors. YY, LL, and JL obtained ethical approval, contributed to the preparation of the data set, interpreted the results, and contributed to drafts of the paper. HW, JW, and JG contributed to the study design and interpretation of the results and commented on drafts of the paper. Specifically, colleagues from the Department of Obstetrics and Gynecology were in charge of the diagnosis, treatment, and follow-up of the patients, while colleagues from the Department of Pathology were in charge of the pathological evaluation and molecular diagnosis.

### Conflict of Interest

The authors declare that the research was conducted in the absence of any commercial or financial relationships that could be construed as a potential conflict of interest.
