# Supplementary material for: Germline and Somatic BRCA1/2 Mutations in 172 Chinese Women With Epithelial Ovarian Cancer
Source: Front Oncol. 2020 Mar 10;10:295. doi: 10.3389/fonc.2020.00295 (PMC7077344; doi:10.3389/fonc.2020.00295)
Supplement: Supplementary file 1 [file Table_1.docx]

Supplement Table 1

Patients with *BRCA1/2* mutations classified as VUS, likely pathogenic or pathogenic. N/A, not available. VUS, variant of unknown signiﬁcance.

| Patient No. | Gene | Nucleotide alterations | Chances of amino acid | Germline/somatic | Function |
| --- | --- | --- | --- | --- | --- |
| F004 | *BRCA2* | c.1568A>G | p.(His523Arg) | Germline | VUS |
| F006 | *BRCA1* | c.4066_4069del | p.(Gln1356Lysfs*9) | Germline | Pathogenic |
| F012 | *BRCA1* | c.3841C>T | p.(Gln1281*) | Germline | Pathogenic |
| F013 | *BRCA2* | c.4049A>G | p.(His1350Arg) | Germline | VUS |
| F013 | *BRCA2* | c.6325G>A | p.(Val2109Ile) | Germline | VUS |
| F015 | *BRCA2* | c.544G>A | p.(Glu182Lys) | Somatic | VUS |
| F016 | *BRCA1* | c.375_376insT | p.(Gln126Serfs*16) | Germline | Pathogenic |
| F020 | *BRCA1* | c.5330C>T | p.(Thr1777Ile) | Germline | VUS |
| F022 | *BRCA1* | c.2138C>G | p.(Ser713*) | Germline | Pathogenic |
| F026 | *BRCA1* | c.5521delA | p.(Ser1841Valfs*2) | Germline | Pathogenic |
| F027 | *BRCA1* | c.1356_1357insAG | p.(Glu453Argfs*23) | Germline | Pathogenic |
| F028 | *BRCA2* | c.6325G>A | p.(Val2109Ile) | Germline | VUS |
| F029 | *BRCA1* | c.3877delG | p.(Ala1293Leufs*14) | Somatic | Pathogenic |
| F032 | *BRCA2* | c.67+1G>C | N/A | Germline | Likely pathogenic |
| F032 | *BRCA2* | c.1964delC | p.(Pro655Glnfs*5) | Somatic | Pathogenic |
| F035 | *BRCA2* | c.2920G>A | p.(Asp974Asn) | Germline | VUS |
| F039 | *BRCA2* | c.8542G>A | p.(Glu2848Lys) | Somatic | VUS |
| F039 | *BRCA2* | c.10101A>T | p.(Glu3367Asp) | Somatic | VUS |
| F045 | *BRCA1* | c.1937G>T | p.(Ser646Ile) | Somatic | VUS |
| F048 | *BRCA1* | c.5251C>T | p.(Arg1751*) | Germline | Pathogenic |
| F050 | *BRCA2* | c.3165_3168del | p.(Asn1055Lysfs*4) | Germline | Pathogenic |
| F051 | *BRCA1* | c.3288_3289delAA | p.(Leu1098Serfs*4) | Germline | Pathogenic |
| F053 | *BRCA1* | c.3257T>G | p.(Leu1086*) | Somatic | Pathogenic |
| F054 | *BRCA2* | c.8989T>C | p.(Tyr2997His) | Somatic | VUS |
| F058 | *BRCA2* | c.1591A>G | p.(Lys531Glu) | Germline | VUS |
| F060 | *BRCA1* | c.4327C>T | p.(Arg1443*) | Somatic | Pathogenic |
| F061 | *BRCA1* | c.686_687delCT | p.(Ser229*) | Germline | Pathogenic |
| F062 | *BRCA2* | c.266C>T | p.(Pro89Leu) | Germline | VUS |
| F062 | *BRCA1* | c.3598C>T | p.(Gln1200*) | Germline | Pathogenic |
| F063 | *BRCA2* | c.5722_5723del | p.(Leu1908Argfs*2) | Germline | Pathogenic |
| F064 | *BRCA1* | c.3448C>T | p.(Pro1150Ser) | Germline | VUS |
| F066 | *BRCA2* | c.1964del | p.(Pro655Glnfs*5) | Somatic | Pathogenic |
| F068 | *BRCA1* | c.1966A>T, | p.(Asn656Tyr) | Germline | VUS |
| F068 | *BRCA1* | c.1465G>T | p.(Glu489*) | Germline | Pathogenic |
| F069 | *BRCA1* | c.4675+1G>A | N/A | Germline | Pathogenic |
| F070 | *BRCA1* | c.5333-1G>A | N/A | Somatic | Pathogenic |
| F072 | *BRCA1* | c.5074G>A | p.(Asp1692Asn) | Germline | Pathogenic |
| F083 | *BRCA1* | c.66dupA | p.(Glu23Argfs*18) | Germline | Pathogenic |
| F086 | *BRCA2* | c.6325G>A | p.(Val2109Ile) | Germline | VUS |
| F087 | *BRCA1* | c.284_288del | p.(Leu95Hisfs*10) | Germline | Pathogenic |
| F094 | *BRCA2* | c.3281del | p.(Lys1094Serfs*10) | Germline | Pathogenic |
| F100 | *BRCA1* | c.80+5G>A | N/A | Germline | Likely pathogenic |
| F102 | *BRCA1* | c.5257A>T | p.(Arg1753*) | Germline | Pathogenic |
| F108 | *BRCA2* | c.1568A>G | p.(His523Arg) | Germline | VUS |
| F109 | *BRCA1* | c.4963delT | p.(Ser1655Leufs*3) | Germline | Pathogenic |
| F113 | *BRCA1* | c.5096G>A | p.(Arg1699Gln) | Germline | Likely pathogenic |
| F115 | *BRCA1* | c.514C>T | p.(Gln172*) | Germline | Pathogenic |
| F116 | *BRCA1* | c.671-6T>G | N/A | Germline | VUS |
| F121 | *BRCA2* | c.8517C>A | p.(Tyr2839*) | Germline | Pathogenic |
| F128 | *BRCA2* | c.1568A>G | p.(His523Arg) | Germline | VUS |
| F132 | *BRCA2* | c.3521G>T | p.(Gly1174Val) | Germline | VUS |
| F133 | *BRCA2* | c.1625_1626del | p.(Ile542Thrfs*17) | Germline | Pathogenic |
| F136 | *BRCA1* | c.66dupA | p.(Glu23Argfs*18) | Germline | Pathogenic |
| F140 | *BRCA2* | c.6325G>A | p.(Val2109Ile) | Germline | VUS |
| F142 | *BRCA1* | c.441+9A>G | N/A | Germline | VUS |
| F144 | *BRCA2* | c.3794G>T | p.(Cys1265Phe) | Germline | VUS |
| F144 | *BRCA1* | c.5470_5477delATTGGGCA | p.(Ile1824Aspfs*3) | Germline | Pathogenic |
| F152 | *BRCA1* | c.981_982delAT | p.(Cys328*) | Germline | Pathogenic |
| F154 | *BRCA1* | c.5470_5477delATTGGGCA | p.(Ile1824Aspfs*3) | Germline | Pathogenic |
| F156 | *BRCA1* | c.5108A>G | p.(Tyr1703Cys) | Germline | VUS |
| F158 | *BRCA2* | c.5073dup | p.(Trp1692Metfs*3) | Germline | Pathogenic |
| F160 | *BRCA2* | c.5682C>G | p.(Tyr1894*) | Germline | Pathogenic |
| F164 | *BRCA2* | c.5242delA | p.(Ser1748Alafs*29) | Germline | Pathogenic |
| F169 | *BRCA2* | c.4218_4221del | p.(Lys1406Asnfs*3) | Germline | Pathogenic |
| F171 | *BRCA2* | c.9182T>G | p.(Leu3061*) | Germline | Pathogenic |
| F173 | *BRCA2* | c.6405_6409del | p.(Asn2135Lysfs*3) | Somatic | Pathogenic |
| F173 | *BRCA1* | c.2286A>T | p.(Arg762Ser) | Somatic | VUS |
