# Supplementary material for: Germline and Somatic BRCA1/2 Mutations in 172 Chinese Women With Epithelial Ovarian Cancer
Source: Front Oncol. 2020 Mar 10;10:295. doi: 10.3389/fonc.2020.00295 (PMC7077344; doi:10.3389/fonc.2020.00295)
Supplement: Supplementary file 2 [file Table_2.docx]

Supplement Table 2

Relationship between survival outcomes and deleterious variants in the overall cohort and in HGSC patients. All hazard ratios were calculated by Kaplan–Meier analysis with no carriers of deleterious variants as references. All the modified hazard ratios were calculated with a Cox regression model adjusted for the risk factors of major histological subtype (high-grade serous carcinoma versus others, in the overall cohort), age at diagnosis, and stages (stage I-II versus III-IV). HGSC, high-grade serous carcinoma. HR, hazard ratio. 95% CI, 95% confidence interval. N/A, not available. OS, overall survival. PFS, progression-free survival.

|  | All *BRCA1/2* | Germline *BRCA1/2* | Somatic *BRCA1/2* | *BRCA1* | *BRCA2* |
| --- | --- | --- | --- | --- | --- |
| Whole cohort (n=172) |  |  |  |  |  |
| Recurrence with aberrations, n/n (%) | 20/41 (48.8%) | 19/35 (54.3%) | 2/7 (28.6%) | 14/28 (50.0%) | 6/13 (46.2%) |
| Recurrence without aberrations, n/n (%) | 86/131 (65.6%) | 86/131 (65.6%) | 86/131 (65.6%) | 86/131 (65.6%) | 86/131 (65.6%) |
| p values | 0.053 | 0.215 | 0.059 | 0.120 | 0.138 |
| Mortality with aberrations, n/n (%) | 8/41 (19.5%) | 8/35 (22.9%) | 1/7 (14.3%) | 6/28 (21.4%) | 2/13 (15.4%) |
| Mortality without aberrations, n/n (%) | 42/131 (32.1%) | 42/131 (32.1%) | 42/131 (32.1%) | 42/131 (32.1%) | 42/131 (32.1%) |
| p values | 0.123 | 0.292 | 0.299 | 0.266 | 0.178 |
| PFS, HR (95% CI) | 0.605 (0.372-0.985) | 0.692 (0.421-1.137) | 0.346 (0.085-1.407) | 0.633 (0.360-1.114) | 0.551 (0.241-1.262) |
| p values | **0.037** | 0.136 | 0.113 | 0.103 | 0.144 |
| Adjusted PFS, HR (95% CI) | 0.570 (0.347-0.933) | 0.647 (0.391-1.068) | 0.305 (0.074-1.254) | 0.603 (0.340-1.072) | 0.480 (0.208-1.111) |
| p values | **0.026** | 0.088 | 0.100 | 0.085 | 0.087 |
| OS, HR (95% CI) | 0.400 (0.187-0.858) | 0.457 (0.213-0.979) | 0.319 (0.044-2.329) | 0.441 (0.186-1.046) | 0.313 (0.075-1.305) |
| p values | **0.014** | **0.038** | 0.230 | 0.054 | 0.089 |
| Adjusted OS, HR (95% CI) | 0.386 (0.179-0.834) | 0.453 (0.210-0.974) | 0.233 (0.031-1.737) | 0.488 (0.049-0.905) | 0.234 (0.055-0.987) |
| p values | **0.015** | **0.043** | 0.155 | 0.108 | **0.048** |
| HGSC patients (n=138) |  |  |  |  |  |
| Recurrence with aberrations, n/n (%) | 17/38 (44.7%) | 16/32 (50.0%) | 2/7 (28.6%) | 13/27 (48.1%) | 4/11 (36.4%) |
| Recurrence without aberrations, n/n (%) | 71/100 (71.0%) | 71/100 (71.0%) | 71/100 (71.0%) | 71/100 (71.0%) | 71/100 (71.0%) |
| p values | **0.004** | **0.029** | **0.032** | **0.026** | **0.026** |
| Mortality with aberrations, n/n (%) | 8/38 (21.1%) | 8/32 (25.0%) | 1/7 (14.3%) | 6/27 (22.2%) | 2/11 (18.2%) |
| Mortality without aberrations, n/n (%) | 34/100 (34.0%) | 34/100 (34.0%) | 34/100 (34.0%) | 34/100 (34.0%) | 34/100 (34.0%) |
| p values | 0.140 | 0.341 | 0.266 | 0.242 | 0.241 |
| PFS, HR (95% CI) | 0.496 (0.292-0.843) | 0.573 (0.333-0.986) | 0.302 (0.074-1.232) | 0.537 (0.297-0.971) | 0.396 (0.145-1.086) |
| p values | **0.010** | **0.044** | 0.095 | **0.040** | 0.072 |
| Adjusted PFS, HR (95% CI) | 0.518 (0.305-0.882) | 0.587 (0.341-1.011) | 0.317 (0.077-1.307) | 0.577 (0.319-1.044) | 0.378 (0.137-1.046) |
| p values | **0.015** | 0.055 | 0.112 | 0.069 | 0.061 |
| OS, HR (95% CI) | 0.488 (0.223-1.066) | 0.572 (0.262-1.250) | 0.317 (0.043-2.329) | 0.524 (0.218-1.259) | 0.396 (0.094-1.666) |
| p values | 0.072 | 0.161 | 0.259 | 0.148 | 0.206 |
| Adjusted OS, HR (95% CI) | 0.468 (0.214-1.027) | 0.562 (0.257-1.229) | 0.238 (0.032-1.778) | 0.547 (0.227-1.320) | 0.310 (0.072-1.326) |
| p values | 0.058 | 0.149 | 0.162 | 0.180 | 0.114 |
